# Association between Periodontal Disease and Oral Benign, Potentially Malignant, Malignant, and Chronic Immune-Mediated Disorders: A Clinical Study

**DOI:** 10.3390/healthcare12191999

**Published:** 2024-10-07

**Authors:** Antonio Barbarisi, Francesca Cremonini, Dorina Lauritano, Valeria Visconti, Gianluigi Caccianiga, Saverio Ceraulo

**Affiliations:** 1 Department of Medicine and Surgery, University of Milano-Bicocca, 20100 Monza, Italy; 2 Fondazione IRCCS San Gerardo dei Tintori, 20900 Monza, Italy; 3 Department of Translational Medicine, University of Ferrara, 44121 Ferrara, Italy; 4 Postgraduate School of Orthodontics, University of Ferrara, 44121 Ferrara, Italy

**Keywords:** periodontal disease, lichen planus, leucoplakia, oral cancer, OHLLT

## Abstract

**Background**: Periodontal disease is an inflammatory, chronic, and multifactorial disease. The objective of this study is to analyze the association between periodontal disease and some disorders such as papillomas (benign lesions), lichen planus (a chronic immune-mediated disorder), leukoplakia (potentially malignant lesions), and oral cancer (malignant lesions). **Methods**: For this study, 42 patients were recruited whose supragingival and subgingival plaque was qualitatively analyzed using a phase-contrast microscope, which allowed for the detection of compatible bacterial flora (immobile and composed mainly of cocci) indicative of periodontal health and incompatible bacterial flora (mobile and composed mainly of spirochetes) indicative of periodontal pathology. Patients with incompatible bacterial flora were then subjected to a laser-assisted periodontal treatment with irrigation with hydrogen peroxide within the periodontal pockets (a non-surgical laser-assisted periodontal protocol which is referred to as dye-free photodynamic therapy). **Results**: Based on the 42 patients recruited, there was no association between oral cavity lesions and periodontal pathogenic bacteria. Four of them were found to have incompatible bacterial flora. Indeed, it was found that almost all the patients had been previously instructed in the proper techniques of home oral hygiene, and more than half of them reported that they carried out periodic check-ups by a dental hygienist. Of the four patients with signs and symptoms of periodontitis, two stated a willingness to undergo the non-surgical laser-assisted periodontal protocol and showed improvements in periodontal indices such as CAL, PPD, and BoP. **Conclusions**: hygienists and dentists are determining factors in the prevention of periodontal disease and for the maintenance of good oral health.

## 1. Introduction

Periodontal disease is a chronic degenerative inflammatory disease that affects the supporting tissues of the teeth and is caused by Gram-negative anaerobic bacteria (i.e., Porphyromonas gingivalis and Tannerella forsythia). These bacterial species adhere to the surface of teeth and form dental plaque, also referred to as biofilm [1,2,3]. This illness is reversable (it is called gingivitis), but if it is not treated, it can degenerate into periodontitis, which is irreversible and leads to bone destruction [4,5]. Periodontitis affects over 50% of the population, indicating a dose–response relationship with oral health and quality of life [6,7,8].

As is evident from the literature, the association between periodontal disease and some oral cavity lesions is well documented, while others require further studies [9]. There is an axis of interaction between Porphyromonas gingivalis, considered to be the main bacterial periodontal pathology, and the herpes simplex virus. The two appear to be etiologic cofactors and trigger relapses in periodontitis [10]. A more recent review states that the herpes virus (a viral disease caused by the herpes simplex virus) and periodontal bacteria have complementary mechanisms of infection, which lead to the progression of periodontal disease [11]. Periodontitis also has a close association with oral lichen planus (OLP), a chronic inflammatory condition caused by an autoimmune disorder (so the immune system mistakenly attacks oral mucosal cells), which particularly affects the tongue, cheeks, palate, and gums. It commonly affects middle-aged females, and the most common symptoms are pain, roughness, and other discomfort [12]. Leukoplakia is a precancerous lesion and is still linked to periodontal disease. It is a condition characterized by the presence of white patches on the mucosa of the mouth: they may appear on the cheeks, the gums, on the tongue, or on the palate. Leukoplakia is a clinical definition, without a histologic correlation: it can be an atrophy, a hyperplasia, or a dysplasia. The evolution of this lesion is variable, and it can transform into a malignant lesion [13].

Periodontal disease is commonly associated with bacteria, but yeasts have also been found in periodontal pockets, such as Candida albicans. This microorganism is found in the oral cavity under normal conditions, and its role in periodontitis has been hypothesized. It appears, in fact, to be responsible for the persistence of the disease (in other words, it is responsible for chronic disease) [14].

Oral papillomatosis is a heteromorphic group of benign lesions of the oral mucosa, and the human papillomavirus (HPV) can be an etiological factor [15]. Non-viral etiologies such as smoking, alcoholism, and the accumulation of bacterial plaque as a result of poor oral hygiene and tartar may also cause oral papilloma lesions [16].

The same etiological factors inducing papillomas are incriminated in the development of malignant tumors of the oral cavity, as part of head and neck squamous cell carcinoma (HNSCC) [17,18], located in the oral cavity, oropharynx, hypopharynx, larynx, and nasopharynx. HPV drives oropharyngeal squamous cell carcinoma (OPSCC), while tobacco and alcohol consumption are responsible for HNSCC in other locations [19,20,21,22].

The primary aim of this study was to verify the presence of an association between patients with diseases of the oral cavity, including oral, preneoplastic, neoplastic, and chronic immune-mediated disorders, and periodontitis from the perspective of microbiology. The secondary target was to treat patients with periodontal bacteria following a periodontal protocol, including the use of the GBT protocol and dye-free photodynamic therapy, evaluating improvements and benefits to patients.

## 2. Materials and Methods

The presence of typical signs of periodontitis in patients with oral disorders, such as papillomas, lichen planus, leukoplakia, and oral cancer, was investigated.

The study was conducted according to the guidelines of the Ethics Committee of the School of Medicine and Surgery at the Milano Bicocca University (protocol n. 11/17) and executed in conformity with the Declaration of Helsinki.

A qualitative analysis was performed on the supragingival and subgingival plaque of the patients examined using a phase-contrast microscope, which was able to confirm or disprove the presence of pathogenic bacteria causing periodontal disease. Patients presenting with incompatible bacterial flora, indicative of significant periodontal disease, underwent a non-surgical laser-assisted periodontal treatment in order to reduce inflammation, promote tissue healing, and improve their overall periodontal health. Patients diagnosed with periodontitis who decided to undergo treatment for this pathology began the non-surgical laser-assisted periodontal protocol called “dye-free photodynamic therapy” (irrigation of periodontal pockets with hydrogen peroxide and irradiation with diode laser with the following settings: 980 nM, peak power of 3 W, ton of 20 micros, toff of 80 micros, and average power of 0.6 W).

### 2.1. Study Design

At T0, after agreeing to join the research project and signing the various consent forms (concerning privacy, consent to receive treatment, and photographic consent), the following information was collected from each patient:

- medical history (health status, diseases, pregnancy, habits, etc.);
- dental check-up (for the purpose of establishing the general condition of the oral cavity);
- oral hygiene status check using the plaque index of O’Leary;
- instructions regarding and motivation for adoption of the home-modified oral hygiene protocol using sonic toothbrush, brush, and oral cleaner;
- periodontal screening (PSR) to assess periodontal status;
- qualitative microbiological analysis of supra- and subgingival plaque (including sampling of supra- and subgingival plaque with a curette);
- analysis using phase-contrast microscopy, which made it possible to distinguish between compatible bacterial flora (immobile flora), indicative of healthy periodontal pathology, and incompatible bacterial flora (mobile flora), indicative of periodontal pathology;
- Patients presenting incompatible bacterial flora in the analysis by phase-contrast microscopy and who were positive on the PSR screening were referred for periodontal evaluation according to the following:
- completion of the periodontal chart;
- probing depth (PPD);
- presence of gingival recessions;
- bleeding index (BoP);
- evaluation of periodontal radiographic status;
- collection of photographic documentation.
- Patients who met the diagnostic criteria for periodontal disease were given a report with a diagnosis of gingivitis or periodontitis (stage and grade) and were invited to start a protocol of periodontal treatment.

### 2.2. Operative Protocol of Dye-Free Photodynamic Therapy

- At T1, the patients underwent nonsurgical periodontal therapy of the first and second quadrants (upper arch) according to the GBT protocol (plaque detection, air flow with glycine and erythritol, ultrasonic instrumentation, which leads to an increase in patient compliance, and the removal of the disclosed plaque to more effectively access visible calculus deposits [23,24]) and dye-free photodynamic therapy.
- At T2, one week after T1, the patients underwent nonsurgical periodontal therapy of the third and fourth quadrants (lower arch) according to the GBT protocol and dye-free photodynamic therapy.
- At T3, three weeks after T2, the patients underwent dye-free photodynamic therapy for both the upper arch and the lower arch.
- At T4, three weeks after T3, the patients underwent dye-free photodynamic therapy for both the upper arch and the lower arch.
- At T5, three weeks after T4, the patients underwent dye-free photodynamic therapy for both the upper arch and the lower arch.
- At T6, four weeks after T5, the patients underwent reassessment of their bacterial flora by phase-contrast microscopic analysis and reassessment of their periodontal indices (PI, PPD, gingival recessions, and BoP).

The expected outcomes at the end of treatment were as follows:

- improvement in oral hygiene;
- presence of compatible bacterial flora;
- improvement and stabilization over time of clinical periodontal parameters (PI, PPD, recessions, and BoP).

### 2.3. Materials and Tools Used

The following were used during the two phases of experimentation:

- phase-contrast microscope (Leica DM1000, Leica Microsystems GmbH, Wetzlar, Germany) to perform microbiological analysis of plaque;
- diode laser (Doctor Smile Wiser 3, LAMBDA SpA, Brendola (VI), Italy) to administer non-surgical laser-assisted periodontal therapy.

### 2.4. Flowchart

**Figure 1:**
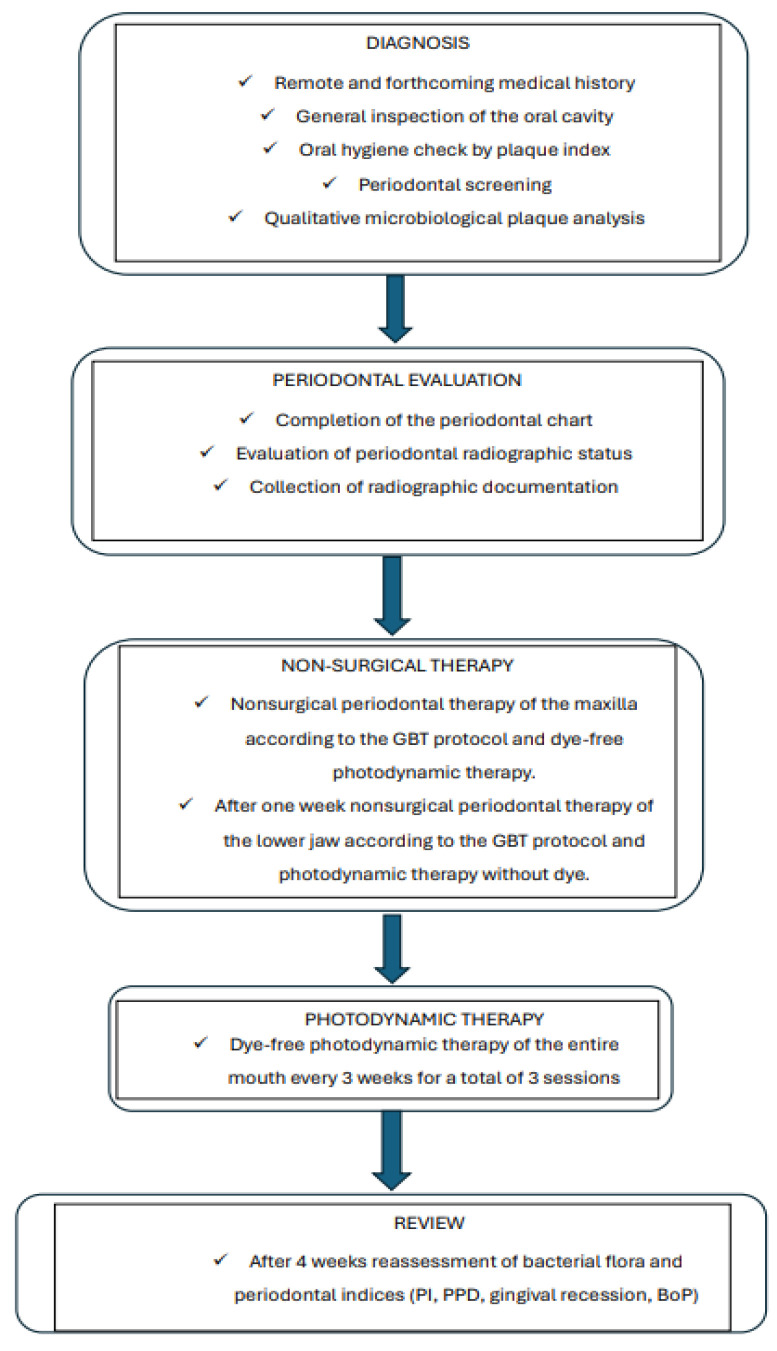
Diagnostic and therapeutic process.

The diagnostic and therapeutic process followed in the study consists of several steps summarized in the flowchart below (Figure 1).

### 2.5. Study Population

The inclusion or exclusion of patients in this study was based on the following factors:

- age (older than 18 years);
- diagnosis as per biopsy examination;
- moving flora (incompatible).

Only subjects with overt lesions (lichen planus, leukoplakia, and oral cancer) were deemed eligible and subsequently were contacted and invited to present themselves for a follow-up examination. Once in the department, during the follow-up visit, they underwent a microbiological examination of their plaque using a phase-contrast microscope (Table 1). The essential inclusion parameter within the study involves the detection of movable (incompatible) flora.

### 2.6. Process of Inclusion and Exclusion

The test population was selected on the basis of patients from whom biopsies were taken at the outpatient clinic for oral pathology at the Dental Clinic of IRCCS San Gerardo dei Tintori in Monza, Italy from the year 2018 to the year 2022.

The histological reports of these patients were reviewed, and this study included only those who were confirmed to have one of the following pathologies: lichen planus, leukoplakia, papillomas, or oral cancer.

## 3. Results

In the year 2018, 28 patients were identified with the following results:

- 12 with leukoplakia;
- 6 with carcinoma;
- 4 with papillomas;
- 6 with lichen planus.

In the year 2019, 19 patients were identified with the following results:

- 9 with leukoplakia;
- 1 with carcinoma;
- 5 with papillomas;
- 4 with lichen planus.

In the year 2020, three patients were identified with the following results:

- 0 with leukoplakia;
- 3 with carcinoma;
- 0 with papillomas;
- 0 with lichen planus.

In the year 2021, three patients were identified with the following results:

- 1 with leukoplakia;
- 0 with carcinoma;
- 0 with papillomas;
- 2 with lichen planus.

In the year 2022, three patients were identified with the following results:

- 0 with leukoplakia;
- 0 with carcinoma;
- 1 with papillomas;
- 2 with lichen planus.

A total of 56 patients over the 5-year period were identified with the following results:

- 22 with leukoplakia;
- 10 with carcinoma;
- 10 with papillomas;
- 14 with lichen planus.

### Preliminary Outcomes of the Sample Search

The 56 patients selected during the inclusion and exclusion process were contacted for the purpose of taking microbiological samples of their plaque. Of the total, 42 patients agreed to provide samples:

- 19 patients with a previous diagnosis of leukoplakia;
- 8 patients with a previous diagnosis of carcinoma;
- 5 patients with a previous diagnosis of papillomas;
- 10 patients with a previous diagnosis of lichen planus.

**Table 1:** Report of the data collected using a phase-contrast microscope. Compatible: immobile, composed mainly of cocci. Incompatible: mobile, composed mainly of spirochetes.

Patient	Sex	Age (y)	Oral Pathology	Location of the Lesion	Flora Outcome
1	M	43	Leucoplakia	Lower lip	Compatible
2	M	51	Squamous Papilloma	Upper lip	Compatible
3	M	64	Lichen Planus	Gingival mucosa	Compatible
4	F	74	Lichen Planus	Buccal mucosa	Compatible
5	M	52	Leucoplakia	Dorsal tongue	Compatible
6	F	69	Leucoplakia	Border tongue	Compatible
7	M	48	Leucoplakia	Gingival mucosa	Compatible
8	M	66	Leucoplakia	Gingival mucosa	Compatible
9	M	67	Lichen Planus	Buccal mucosa	Compatible
10	M	56	Squamous Cell Carcinoma	Ventral tongue	Compatible
11	M	49	Squamous Papilloma	Gingival mucosa	Incompatible
12	F	64	Leucoplakia	Dorsal tongue	Compatible
13	M	46	Leucoplakia	Buccal mucosa	Compatible
14	M	63	Squamous Cell Carcinoma	Ventral tongue	Compatible
15	M	64	Lichen Planus	Buccal mucosa	Compatible
16	M	53	Leucoplakia	Border tongue	Compatible
17	F	47	Lichen Planus	Buccal mucosa	Compatible
18	F	72	Leucoplakia	Gingival mucosa	Compatible
19	F	49	Leucoplakia	Buccal mucosa	Compatible
20	F	55	Leucoplakia	Buccal mucosa	Compatible
21	F	62	Lichen Planus	Ventral tongue	Incompatible
22	M	51	Leucoplakia	Buccal mucosa	Compatible
23	F	69	Leucoplakia	Buccal mucosa	Compatible
24	M	51	Lichen Planus	Ventral tongue	Compatible
25	M	47	Leucoplakia	Lower lip	Compatible
26	M	44	Squamous Cell Carcinoma	Dorsal tongue	Compatible
27	F	59	Leucoplakia	Dorsal tongue	Incompatible
28	F	57	Squamous Cell Carcinoma	Ventral tongue	Compatible
29	M	60	Squamous Papilloma	Dorsal tongue	Compatible
30	M	39	Leucoplakia	Lower lip	Compatible
31	M	73	Leucoplakia	Lower lip	Compatible
32	M	57	Squamous Papilloma	Buccal mucosa	Compatible
33	F	65	Leucoplakia	Buccal mucosa	Compatible
34	M	42	Squamous Cell Carcinoma	Soft palate	Compatible
35	F	39	Squamous Cell Carcinoma	Ventral tongue	Compatible
36	M	48	Squamous Cell Carcinoma	Ventral tongue	Compatible
37	M	55	Leucoplakia	Border tongue	Incompatible
38	M	50	Squamous Cell Carcinoma	Dorsal tongue	Compatible
39	M	63	Lichen Planus	Buccal mucosa	Compatible
40	F	48	Lichen Planus	Ventral tongue	Compatible
41	F	59	Squamous Papilloma	Gingival mucosa	Compatible
42	F	59	Lichen Planus	Buccal mucosa	Compatible

Out of the total sample:

- 90.5% of the patients (n°38) had compatible flora, while 9.5% of the patients (n°4) had incompatible flora;
- Thirty-three patients reported that they were advised by a medical pathologist or a dentist to get in touch with a professional oral hygienist and implement a thorough oral hygiene protocol at home;
- Nine patients reported that they were not followed up with periodically by a dental hygienist and that they were not motivated to implement a proper home oral hygiene protocol.

Of the 9 patients who reported not having a thorough home oral hygiene protocol, 4 demonstrated incompatible bacterial flora. Two of them decided to undergo therapy following the operative protocol of the dye-free photodynamic therapy described above (Table 2) and an improvement in their condition was observed.

## 4. Discussion

The association between periodontitis and systemic conditions has been debated in the literature. Cardiovascular diseases are strongly associated with periodontal disease: the significant number of similar oral bacterial DNA species found in cardiac samples of patients with periodontitis compared to edentulous patients suggests that the presence of these microorganisms is associated with a direct mechanism, due to the increased vascular bed as a result of the inflammation that is typical of periodontal pockets [25]. Oral factors, especially the number of remaining teeth and the presence of periodontal disease, increase the risk of cardiovascular events [26].

Diabetes mellitus and periodontal diseases correspond to inflammatory diseases that have pathogenic mechanisms in common: most studies have confirmed the association between DM1 and PD [27]. The prevalence and severity of PD are higher in DM1 patients than in healthy subjects.

Periodontitis is also linked to some lesions of the oral cavity [9,10,11,12,13,14,15,16,17,18,19,20,21,22].

The results of the plaque analysis showed that almost all of the patients with a previous diagnosis of leukoplakia, papillomas, carcinoma, or lichen planus in this study had compatible bacterial flora (immobile bacteria; 90.5% of the patients). This result deviated from what has been shown in the literature.

In fact, by analyzing and comparing the periodontal indices of subjects with OLP with healthy subjects, an increase in these values could be found in patients with OLP [28]. In addition, it was verified that patients with OLP have higher levels of infection with Porphyromonas gingivalis, Tannerella forsythia, and Treponema denticola [29]. Leukoplakia also appears to be related to periodontitis, although more data are needed to clarify this association. To date, there seems to be a bidirectional relationship in which patients with leukoplakia are more likely to have loose tooth attachment, and periodontal disease itself can be classified as a risk factor for the occurrence of leukoplakia [30].

The interaction between periodontal bacteria and oral cancer has been demonstrated. These microorganisms contribute to the progression of neoplasia at various levels (cell survival, apoptosis, proliferation, and invasion), either by a direct bacterial effect or an indirect inflammatory response [31].

In this study, by analyzing the oral hygiene of each patient, we attempted to find commonalities. In fact, the participants in the population, following their diagnoses, were instructed by the pathologist to maintain optimal oral health by improving their hygiene in order to preserve a state of eubiosis and moderate the proliferation of pathogens.

From the observational study conducted, it was found that the patients who were treated and motivated by a team of dental hygienists, after their diagnosis and treatment, had a reduced but healthy periodontium and compatible flora bacteria, in contrast to the findings in the literature. In contrast, the patients who were found to have incompatible bacterial flora stated that they received no guidance from their oral pathologist on how to improve their oral hygiene at home, and they engaged less frequently in preventive tertiary care with a dental hygienist. From the results obtained, therefore, it can be said that the association between benign, potentially malignant, malignant, and chronic immune-mediated oral disorders and periodontal disease is not so clear, as reported in the literature [12,13,15,16,17,18,19,20,21,22].

It should be noted, however, that in this research, proper oral hygiene at home and at work was considered as a factor, which was not considered in the previous studies analyzed, but which proved to be fundamental. Thus, one can trace the presence of compatible flora and the absence of periodontal disease in the patients who were analyzed as having good oral hygiene. A further confirmation of this can be seen in that the patients who were found to have periodontal disease stated that they did not attend periodic sessions with a dental hygienist. They then underwent a protocol of professional oral hygiene therapy and dye-free photodynamic therapy, which resulted in improvements in their periodontal indices (CAL, PPD, and BoP).

In fact, in periodontology, a laser (light amplification by stimulated emission of radiation) is strongly recommended for non-surgical treatment of chronic periodontitis [32]. This instrument is used to induce a bio-stimulatory effect on cells, a vasodilator effect on microcirculatory vessels, and a decontaminating effect on microbial components, which are essential for clot formation, protection, and maturation (which are essential for the purpose of achieving tissue regeneration) [33]. There are many lasers used in dentistry, and they differ from each other in terms of the medium that powers them. Erbium, CO_2_, and diode lasers are the most common. Studies show that diode and erbium lasers are more efficient than CO_2_ lasers because, unlike the latter, they have a limited extent of thermal damage [34]. It is crucial to emphasize that different types of lasers have different effects, depending on the target and their wavelength.

Just for this reason, diode lasers are preferable to erbium lasers and CO_2_ lasers, since they have the ability to penetrate areas deeply. In fact, if the transmitted energy is absorbed and consequently does not penetrate an area, there will be no bio-stimulating effects. Studies show that the growth and differentiation of osteoblasts, which are responsible for bone formation and regeneration, are enhanced by treatment with 940 nm diode lasers [35].

Using the LANAP (laser-assisted new attachment procedure) technique can achieve excellent results, but the optical fiber determines the extent of the thermal overheating of the affected area [36]. OHLLT (oxygen high-level laser therapy) can overcome the limitations of LANAP and improve the effectiveness of the treatment itself by using a colorless substance as an intermediary (dye-free photodynamic therapy). Hydrogen peroxide (H2O2) is used to irrigate the periodontal pockets before proceeding with the treatment. It is essential that this substance is colorless because otherwise it would reduce the penetration capacity of the radiation of the medical lasers used [37].

PDT has emerged as a beneficial treatment option for periodontitis. Combined with SRP, it has a slight advantage in the treatment of periodontitis [38], and there is a short-term benefit when using PDT in addition to SRP in terms of clinical outcome variables.

Additionally, for dental implants, the use of dye-free photodynamic therapy plays a central role in improving clinical outcomes. Studies in fact show that this type of bacterial decontamination is not achievable with other treatment modalities, such as using only manual instrumentation, antibiotic therapy, or LLLT (low-level laser therapy) [39].

The two patients with periodontitis and periodontopoatogenic bacteria present who were treated with the proposed protocol had an improvement in their condition. Both had severe periodontitis, stage IV and grade B, and after 6 weeks, not only had a stabilization of the disease been observed, but also an improvement in the clinical periodontal parameters of CAL, PPD, and BoP, as well as their home oral hygiene habits. Therefore, this protocol made it possible to improve (within the limits of the patients’ initial critical situations) the conditions of these two patients. During the patients’ re-evaluation, periodontal pockets were still present and bleeding on probing still occurred, indicating they may be candidates for subsequent surgical periodontal therapy.

The protocol drawn up for both the diagnostic and therapeutic parts of this study can be applied by dental hygienists, as it is within their skillset.

## 5. Conclusions

From our study conducted on 42 patients with oral cavity disorders, it was shown that there was no association between benign, potentially malignant, malignant, and chronic immune-mediated disorders and periodontal disease.

This shows that hygienists and dentists are determining factors in the prevention of periodontal disease and for the maintenance of good oral health. This conclusion was reached because, from the very beginning, the trial produced different results than expected. What is common amongst most of the subjects in the examined population is the treatment course following their diagnosis: periodic check-ups every 6 months, periodic appointments with a professional oral hygienist, the receipt of instructions regarding home oral hygiene protocols, and being motivated to maintain home oral hygiene protocols. Further studies with larger samples are needed to confirm the obtained results.

## Figures and Tables

**Table 2:** Report of data collected from 2 patients after dye-free photodynamic therapy.

Patient	Staging	Grading	Mean CAL at T0	Mean PPD at T0	%BoP at T0	Mean CAL at T6	Mean PPD at T6	%BoP at T6
1	IV	B	10 mm	6 mm	89%	9 mm	5 mm	25%
2	IV	B	10 mm	8 mm	94%	8 mm	6 mm	28%

## Data Availability

Data from this study are available upon reasonable request by writing to the corresponding.
